# Supplementary material for: Predicting the Potential Current and Future Distribution of the Endangered Endemic Vascular Plant Primula boveana Decne. ex Duby in Egypt
Source: Plants (Basel). 2020 Jul 29;9(8):957. doi: 10.3390/plants9080957 (PMC7463592; doi:10.3390/plants9080957)
Supplement: Supplementary file 1 [file plants-09-00957-s001.pdf]

Supplementary Materials (Appendices)

Table S1. A Pearson correlation matrix among measured population parameters of *P. boveana*. Bold values are significantly different at $p < 0.05$.

Variables	Density	Cover	Size Index	Plant Vigor
Density	1	0.516	**0.798**	0.044
Cover		1	0.208	0.462
Size index			1	0.428
Plant vigor				1

Table S2. Environmental datasets used in the analysis.

Type	Code/Unit	Environmental Variables	Source and Resolution
Bioclimatic	Bio1 (°C)	Annual mean temperature	WorldClim v.2 (~1 km^2)
	Bio2 (°C)	Mean diurnal range (max. temp–min. temp)	
	Bio3 (°C)	Isothermality (Bio2/Bio7) × 100	
	Bio4 (unitless)	Temperature seasonality (SD × 100)	
	Bio5 (°C)	Max temperature of warmest month	
	Bio6 (°C)	Min temperature of coldest month	
	Bio7 (°C)	Temperature annual range (Bio5-Bio6)	
	Bio8 (°C)	Mean temperature of wettest quarter	
	Bio9 (°C)	Mean temperature of driest quarter	
	Bio10 (°C)	Mean temperature of warmest quarter	
	Bio11 (°C)	Mean temperature of coldest quarter	
	Bio12 (mm)	Annual precipitation	
	Bio13 (mm)	Precipitation of wettest month	
	Bio14 (mm)	Precipitation of driest month	
	Bio15 (unitless)	Precipitation seasonality (Coefficient of variation)	
	Bio16 (mm)	Precipitation of wettest quarter	
	Bio17 (mm)	Precipitation of driest quarter	
	Bio18 (mm)	Precipitation of warmest quarter	
	Bio19 (mm)	Precipitation of coldest quarter	
	Thermicity index (°C)	Sum of mean annual temp., min. temp. of coldest month, max. temp. of the coldest month, × 10	ENVIREM (~1 km^2).
	PET seasonality (mm/month)	Potential evapotranspiration (PET)	
	Annual PET (mm/year)	A measure of the ability of the atmosphere to remove water through evapotranspiration	
	PET wettest quarter (mm/month)	Mean monthly PET of wettest quarter	
	PET warmest quarter (mm/month)	Mean monthly PET of warmest quarter	
	Continentality (°C)	Average temp. of warmest month-average temp. of coldest month	
	ClimMoisIndex (unitless)	A metric of relative wetness and aridity	
	Aridity index (unitless)	The degree of water deficit below water need	
Topographic	Elev (m. a.s.l)	Elevation	DIVA-GIS (1 × 1 km)
	Slope (%)	Slope	Derived from Elev.
	Aspect (degree)	Aspect	Derived from Elev.
Edaphic *	BD (g/cm^3)	Bulk density	SoilGrids (1 km^2)
	CF (%)	Coarse fragments volumetric	
	Clay (%)	Clay content	
	Silt (%)	Silt content	
	Sand (%)	Sand content	
	CEC (cmolc/kg)	Cation exchange capacity	
	OC (g/kg)	Organic carbon content	
	pH	pH in H_2O	

*Edaphic data are mean of four soil depths interval (0.00, 0.05, 0.15 and 0.30 m).
